# Pharmacokinetics of Chlorin *e*_6_-Cobalt Bis(Dicarbollide) Conjugate in Balb/c Mice with Engrafted Carcinoma

**DOI:** 10.3390/ijms18122556

**Published:** 2017-11-28

**Authors:** Arthur B. Volovetsky, Vladimir S. Sukhov, Irina V. Balalaeva, Varvara V. Dudenkova, Natalia Yu. Shilyagina, Аlexey V. Feofanov, Anastasija V. Efremenko, Mikhail A. Grin, Andrey F. Mironov, Igor B. Sivaev, Vladimir I. Bregadze, Anna V. Maslennikova

**Affiliations:** 1 Department of Biophysics, Lobachevsky State University of Nizhny Novgorod, 23 Gagarina Av., Nizhny Novgorod 603950, Russia; vssuh@mail.ru (V.S.S.); irin-b@mail.ru (I.V.B.); orannge@mail.ru (V.V.D.); nat-lekanova@yandex.ru (N.Y.S.); maslennikova.anna@gmail.com (A.V.M.); 2 Department of Oncology, Nizhny Novgorod State Medical Academy, 10/1 Minin and Pozharsky Sq., Nizhny Novgorod 603005, Russia; 3 Shemyakin and Ovchinnikov Institute of Bioorganic Chemistry, Russian Academy of Sciences, 16/10 Miklukho-Maklaya Str., Moscow 117997, Russia; avfeofanov@yandex.ru (A.V.F.); aefr@mail.ru (A.V.E.); 4 Biological Faculty, Lomonosov Moscow State University, Vorobyevi Gori 1, Moscow 119992, Russia; 5 Institute of Fine Chemical Technology, Moscow Technological University, 86 Vernadskii Av., Moscow 119571, Russia; michael_grin@mail.ru (M.A.G.); mironov@mitht.ru (A.F.M.); 6 Nesmeyanov Institute of Organoelement Compounds, Russian Academy of Sciences, 28 Vavilov Str., Moscow 119991, Russia; sivaev@ineos.ac.ru (I.B.S.); bre@ineos.ac.ru (V.I.B.)

**Keywords:** boron neutron capture therapy, fluorescent microscopy, boron content, MS-ICP, simple multichamber model, chlorin *e*_6_ derivatives

## Abstract

The necessary precondition for efficient boron neutron capture therapy (BNCT) is control over the content of isotope ^10^B in the tumor and normal tissues. In the case of boron-containing porphyrins, the fluorescent part of molecule can be used for quantitative assessment of the boron content. **Study Objective:** We performed a study of the biodistribution of the chlorin *e*_6_-Cobalt bis(dicarbollide) conjugate in carcinoma-bearing Balb/c mice using ex vivo fluorescence imaging, and developed a mathematical model describing boron accumulation and release based on the obtained experimental data. **Materials and Methods:** The study was performed on Balb/c tumor-bearing mice (CT-26 tumor model). A solution of the chlorin *e*_6_-Cobalt bis(dicarbollide) conjugate (CCDC) was injected into the blood at a dose of 10 mg/kg of the animal’s weight. Analysis of the fluorescence signal intensity was performed at several time points by spectrofluorimetry in blood and by laser scanning microscopy in muscle, liver, and tumor tissues. The boron content in the same samples was determined by mass spectroscopy with inductively coupled plasma. **Results:** Analysis of a linear approximation between the fluorescence intensity and boron content in the tissues demonstrated a satisfactory value of approximation reliability with a Spearman’s rank correlation coefficient of *r* = 0.938, *p* < 0.01. The dynamics of the boron concentration change in various organs, calculated on the basis of the fluorescence intensity, enabled the development of a model describing the accumulation of the studied compound and its distribution in tissues. The obtained results reveal a high level of correspondence between the model and experimental data.

## 1. Introduction

Boron neutron capture therapy (BNCT) is a binary method for the treatment of cancer which is based on the nuclear reaction of two essentially nontoxic species: non-radioactive ^10^B isotope and low-energy thermal neutrons. The neutron capture by ^10^B produces an α-particle, ^4^He^2+^, and ^7^Li^3+^ ion together with 2.4 MeV of kinetic energy and 480 keV photon. These high-linear-energy transfer ions dissipate their kinetic energy before traveling one cell diameter (5–9 μm) in biological tissues, ensuring their potential for precise cell-killing [1,2,3,4,5,6,7,8,9]. Boron determination in blood and tissue samples is a crucial task especially for treatment planning, preclinical research, and clinical application of BNCT [10,11,12]. Since ^10^B isotope is non-radioactive and cannot serve as a mark point for quantitative analysis, an important aspect is the development of reliable methods for non-invasive determination of boron content in vivo. Presently, there are different ways to assess boron accumulation in tissues ex vivo, of which inductively coupled plasma mass spectroscopy (ICP-MS) and atomic emission spectrometry (ICP-AES) [13,14,15], as well as prompt gamma-ray spectroscopy (PGRS) [16,17,18], are the most widely used. Further application of PGRS for BNCT includes the possibility of boron determination in vivo during the treatment of the patient [19,20,21]. Other opportunities for quantitative boron determination in vivo include magnetic resonance imaging (MRI) [22,23,24,25,26] and positron emission tomography (PET) [27,28,29,30].

In the case of the use of fluorescent boron-containing compounds for BNCT, the fluorescence may be used for the quantitative assessment of boron content in biological tissues. The methodological basis for such an approach is the assumption that the intensity of fluorescence of the compound corresponds to the amount of boron contained in the respective tissue sample. This approach has been implemented for the determination of *p*-boronophenylalanine (BPA) content in blood [31], as well as for the study of the biodistribution of boronated porphyrins in small animal models [32,33,34,35].

Recently, we found that the chlorin *e*_6_-Cobalt bis(dicarbollide) conjugates can accumulate selectively in human lung adenocarcinoma A549 cells in concentrations suitable for BNCT [36,37]. The in vivo study demonstrated an increased accumulation of the conjugate with boron nanoparticles in the tumor as compared with normal tissues [38]. An accumulation assessment was performed by determining the intensity of the fluorescenсe signal in samples of biological tissues. There is an issue to address: how accurately does the level of conjugate fluorescence reflect the key indicator of the possibility to use the medication for BNCT (namely, the boron content in tissues)?

Considering the methodological complexity of experimental assessment of the dynamics of boron content in tissues for the purposes of BNCT, the use of a preliminary theoretical assessment by means of a mathematical simulation becomes relevant. At the present time, mathematical models based on the description of pharmacokinetics are widely used in medicine for a preliminary assessment of the necessary medication doses, as well as for the implementation of personalized therapy [39,40]. In particular, such simulations are actively used when developing schemes of chemotherapy [41,42]. It is important to note that the use of pharmacokinetic simulation enables one to abandon a great number of measurements in vivo and ex vivo and provide an evaluation of the compound content at any moment. The method is based on kinetic equations that describe the dynamics of accumulation and subsequent elimination of medications for various organs [43].

The development of pharmacokinetic models is particularly relevant for new medications whose biodistribution and accumulation in tumor tissue is insufficiently studied. In our case, the study of new boron-containing compounds for the purposes of BNCT is of particular interest.

**Figure 1:**
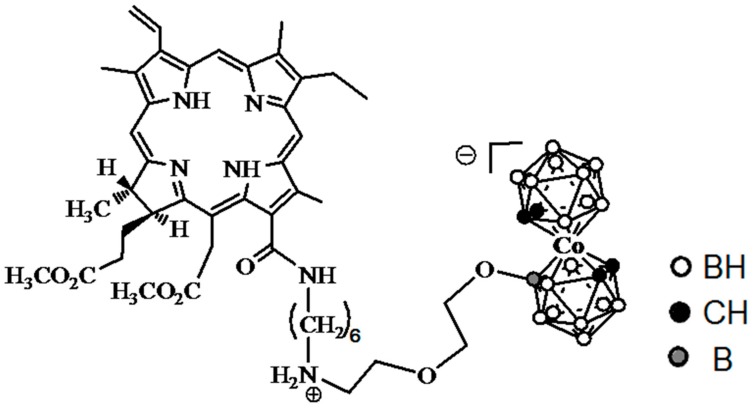
Structure of the studied chlorin *e*_6_-Cobalt bis(dicarbollide) conjugate (CCDC).

***Study objective:*** To study the biodistribution of the chlorin *e*_6_-Cobalt bis(dicarbollide) conjugate (CCDC) (Figure 1) in carcinoma-bearing Balb/c mice using ex vivo fluorescence imaging, and to develop a mathematical model describing boron accumulation and release based on the obtained experimental data.

## 2. Results

**Figure 2:**
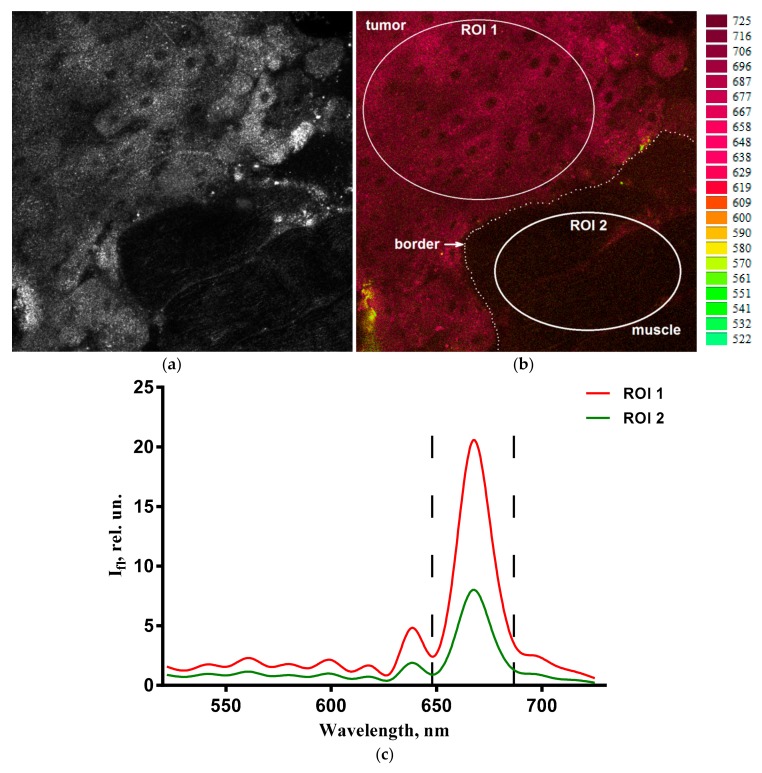
An example of the selection of homogeneous regions of interest (ROIs) in a fluorescence image of a tumor growing into the muscular tissue. (**a**) Reflected light image; (**b**) spectral image of the same area. Image size: 354 × 354 μm. Dashed line depicts the boundary between tumor cells and muscle fibers that was identified using morphological features observed in the reflected light image of the scanned area. (**c**) Fluorescence spectra in ROIs selected in panel (**b**). Dashed lines in panel (**c**) restrict the 648–687 nm range that was used to calculate fluorescent intensity of CCDC in ROIs.

**Figure 3:**
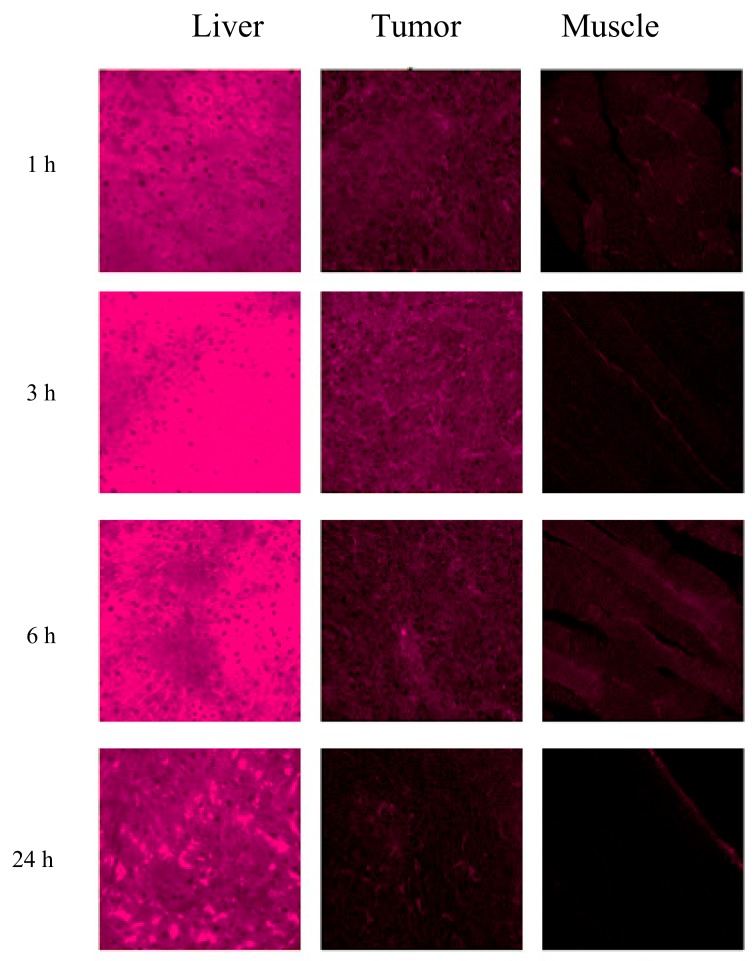
Fluorescent images of animal’s tissue samples in 1, 3, 6, and 24 h after the CCDC injection at a dose of 10 mg/kg. λ_ex_ = 514 nm. λ_em_ = 648–687 nm. Image size is 354 × 354 μm.

When studying samples of organs and tissues of animals by laser scanning microscopy, the maximum fluorescence of CCDC was registered in the 665–670 nm wavelength range (Figure 2), which corresponds to CCDC spectral characteristics in a lipid-like environment [37,38]. The levels of CCDC accumulation in tumor, liver, and muscle tissues were noticeably different (Figure 3). They changed over time and achieved maxima 3 h after the CCDC injection (Figure 3). The highest level of CCDC accumulation was observed in the liver, and the lowest one—in the muscle. Accumulation of CCDC in the tumor tissue was considerably higher than in the muscle (Figure 3) including those that neighbored tumor tissue (Figure 2). Almost complete excretion of CCDC from the muscles and tumor tissue occurred in the 24 h after injection.

**Figure 4:**
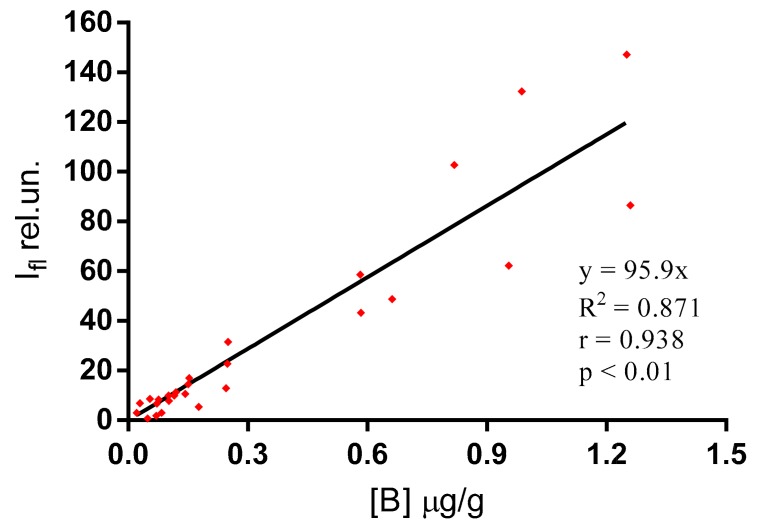
Correlation analysis of the CCDC fluorescence intensity (Ifl) and boron concentration [B] in the studied tissues. Joined data obtained for tumor, muscle, and liver are presented. Spearman’s rank correlation coefficient *r* = 0.938; significance level *p* < 0.01.

To confirm the hypothesis of correspondence between the fluorescent intensity and boron concentration in tissues, the boron content was studied using the ICP-MS method. A comparison of the results obtained by both methods revealed a high positive correlation between the analyzed parameters (Figure 4). Linear fitting of the experimental data provided a satisfactory value of approximation reliability (R^2^ > 0.8), and high Spearman’s rank correlation coefficient (*r* = 0.938) and significance level (*p* < 0.01). This result indicates the stability of the conjugate in blood and tissues for a duration of at least 24 h, and allows one to use the CCDC fluorescence intensity as a proxy indicator of the boron concentration in tissues.

**Figure 5:**
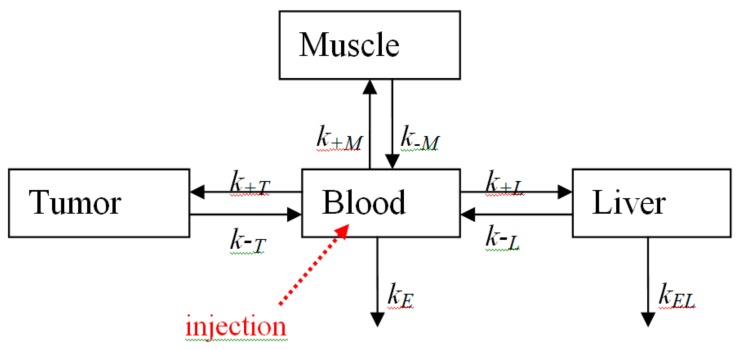
Scheme of a simple multichamber model.

A detailed analysis of the dynamics of boron distribution in tumor and normal tissues was performed with the use of a mathematical simulation method. When building a multichamber mathematical model, we considered the blood, liver, muscle, and tumor as chambers (Figure 5). Blood was assumed to be the primary tissue that interacted with CCDC, transporting it through the organism and participating in CCDC elimination. The liver was considered to be the main depository of CCDC, and its elimination channel [44,45]. Muscle was selected as a sample of a normal tissue.

**Figure 6:**
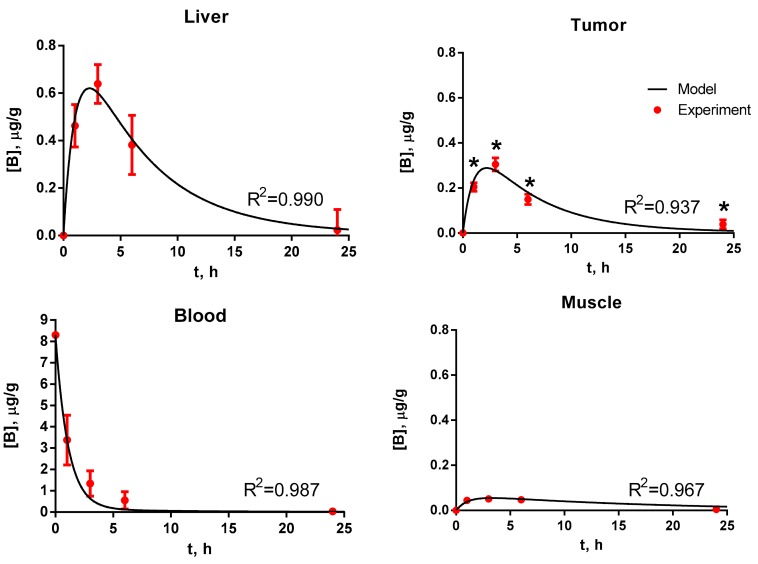
Pharmacokinetics of CCDC injected into the blood at a dose of 10 mg/kg. Dots denote experimental data. Lines denote the results of the solution of the mathematical model.

Using experimentally proved linear dependence between the CCDC fluorescence intensity and the boron concentration (Figure 4), the measured values of fluorescence intensities in tissues were recalculated as boron concentrations for all temporal points. The boron concentration in blood was calculated on the basis of the known amount of the element in the injected compound (8.3 μg/g of blood). A solution to the mathematical model was found by varying the rate constants *k* (Figure 5) until the best correspondence of the model to the experimental data was achieved (Figure 6). The best-fit values of *k* are presented in Table 1. The calculated values of the determination coefficients (Figure 6) prove a high level of correspondence of the model to the experimental data.

Stars mark values of boron concentrations that are significantly higher in the tumor tissue as compared with the muscles (*p* < 0.01).

## 3. Discussion

The issue of the compound stability “on the way” to the target is of great importance when any kind of drug transporter is under development. Analysis of tissue distribution, accumulation, and elimination of boron is accompanied by certain difficulties caused by the fact that B^10^ is not radioactive and cannot be used as a quantitative mark point [1,3]. Results of our study demonstrate a direct correlation between boron concentration and fluorescence of CCDC in different tissues including those of tumors, and this correlation is high (*r* > 0.9) and significant (*p* < 0.01). This indicates the stability of the compound in the tissue during the entire observation time. Using the chlorin *e*_6_ cobalt bis(dicarbollide) conjugate, the necessary (3-2:1) contrast of accumulation of the compound in tumor and muscle was achieved. We assume that the high contrast between tumor and muscle may be due to the enhanced permeability and retention of nano-sized objects in tumors (the so-called EPR-effect) [46]. This effect results from peculiarities of the tumor blood vessel architectonics and a lack of lymphatic drainage. Yet, the decomposition of CCDC in an organism, and its clearance, needs further detailed study.

Since chlorin *e*_6_ derivatives like CCDC absorb light and fluorescence in the spectral range of 650–690 nm, where tissues have increased transparency to light [47], an opportunity arises for indirect determination of boron content in tissues by fluorescent methods in vivo [48]. These methods have definite limitations, because the depth of light penetration in tissues is rather limited and depends on the scattering/absorptive properties of tissues, but developments and applications of photosensitizers for anticancer photodynamic therapy proved that in vivo fluorescence microscopy may be successfully used in the case of small superficially localized tumors [49]. Moreover, a non-invasive fluorescence-based determination of concentrations of CCDC and similar boron conjugates in patients’ blood seems to be possible in large superficial veins [50]. Data on the variations of boron content in tumor and adjacent normal tissues obtained with fluorescence analysis open an easy way to optimize the delay between drug injection and a BNCT procedure, and, thereby, increase the safety, selectivity, and efficiency of the therapy [51,52].

Data on the dynamics of boron concentration in various organs, calculated on the basis of the fluorescence intensity, enabled us to develop a reliable model describing the accumulation of CCDC and its distribution in tissues. This model may become a convenient tool for forecasting dynamics of boron concentration in various organs in subsequent preclinical studies of CCDC and/or similar conjugates [53].

Further development and improvement of the mathematical model may be related to, first, its expansion to other organs, and, second, detailed analysis of the influence of the administration modes of the studied compounds (dose variations, injection portions, inoculation area).

## 4. Materials and Methods

The biodistribution of CCDC containing 18 boron atoms per molecule (Figure 1) was studied. The CCDC was synthesized as described earlier [54]. The purity of the CCDC was 96–98% according to HPLC analysis. Contamination of the CCDC with either free chlorin e6 or free cobalt bis(dicarbollide) was low, and could not lead to noticeable errors in our measurements.

Experiments were carried out on Balb/C mice (female, 6 weeks, 19 ± 1 g) kept in standard vivarium conditions in accordance with the requirements of regulations governing the implementation of research on the safety and efficacy of pharmacological agents in Russia (Order of the Ministry of Health of Russia “On approval of rules of good laboratory practice”), and the international rules of the legal and ethical use of animals. The study was approved by the Research Ethics Board of Lobachevsky University. The CT-26 line of murine large bowel carcinoma was used to obtain experimental tumors. The experiment began on the 9–10th day after inoculation of tumor cells (1 million cells in 100 μL of PBS) into the hip when the tumor node diameter amounted to ~9 mm. In the experimental group (17 animals), the CCDC solution in 5% сremophor emulsion CrEl and 0.9% sodium chloride was injected into the tail vein at a dose of 10 mg/kg of the animal’s weight. The dose was chosen on the basis of data on the concentration providing visualization of the drug in tissues by optical methods [40].

The injection volume was calculated separately for each animal based on its weight and amounted to 90–100 μL. We used intact animals as a control group (3 animals).

For a fluorescent analysis of CCDC in plasma, blood (25 μL) was sampled from the retro-orbital sinus of five animals before injection as well as 5 min, 1, 3, 6, and 24 h after the CCDC injection. Plasma was obtained by blood centrifugation (700× *g* for 5 min), and spectrofluorimetric study was performed using a Shimadzu RF-5301PC spectrofluorometer (Shimadzu, Canby, OR, USA). Fluorescence was excited at the 505 nm wavelength. A signal was detected in the 650–690 nm wavelength range.

Analysis of the fluorescence intensity and boron content was performed in samples of muscle, liver, and tumor tissues for 3 control and 12 experimental animals at 1, 3, 6, and 24 h after CCDC injection (3 animals per each temporal point). Organ samples were divided into two parts for analyses by ICP-MS and laser scanning microscopy methods. Blood samples drawn 3 h after the CCDC injection were used for the ICP-MS analysis.

The samples for fluorescent microscopy and MS-ICP were obtained simultaneously. We took two samples from each organ immediately after euthanizing the animal: one sample for fluorescent analysis, another for MS-ICP. All samples were frozen and stored at −20 °C until required.

The samples were analyzed by confocal microscopy without formalin fixation procedures or staining. An entire tumor was cut from the surrounding tissue and dissected along a longitudinal axis perpendicular to the thigh surface. The femoral muscle was cut along the fibers. The liver was sectioned deep longitudinally in the coronal plane. An earlier pilot study demonstrated that fluorescence signal intensity does not change after freezing. The images were recorded in standard conditions [40] using a confocal laser scanning microscope Axio Observer Z1 LSM 710 DUO (Carl Zeiss, Oberkochen, Germany) with excitation of CCDC fluorescence at the 514 nm wavelength and registration of a spectral signal within the 522–725 nm range with a step size of 10 nm.

A channel corresponding to the fluorescence signal of CCDC within the 648–687 nm range was singled out in the obtained spectral images by means of microscope software [55], and signal intensities were averaged over 2–4 regions of interest (ROIs) corresponding to tumor and normal tissues in each sample image using the ImageJ program (National Institutes of Health, Rockville, MD, USA). Homogeneous (according to the fluorescence signal recorded from sample tissues) ROIs were defined in images without overlapping, as shown in Figure 2. The size of the ROIs varied between 0.5 × 10^5^ and 1.3 × 10^5^ pixels. In total, 223 organ images were processed.

The analysis of boron content in the preserved tissue samples (50–100 μg of muscle, liver, and tumor) was performed in LTD “Micronutrients” (Moscow, Russia) by ICP-MS using the quadrupole mass-spectrometer Nexion 300D (Perkin Elmer, ‎Waltham, MA, USA).

Correlation analysis was performed between the intensity of fluorescence determined by an optical microscopy method and the boron concentration measured by ICP-MS method for all the studied organs and tissues (muscle, liver, tumor). A correlation cloud was created and linearly approximated using the Prism software (GraphPad Software Inc., La Jolla, CA, USA). The Spearman’s rank correlation coefficient and the significance level were calculated.

## 5. Mathematical Simulation

For the analysis of the CCDC dynamics, a simple multichamber model was used [43]. In this model, blood and studied organs (liver, muscles, tumor) (Figure 5) were considered as different chambers.

Such a model was described by a system of differential Equations (1):

(1) $$\frac{\mathrm{dB}}{\mathrm{dt}}=k_{-L}L-k_{+L}B+k_{-M}M-k_{+M}B+k_{-T}T-k_{+T}B{-k}_{E}B\;\frac{\mathrm{dL}}{\mathrm{dt}}=\left(k_{-L}L-k_{+L}B-\;k_{\mathrm{EL}}L\right)\frac{V_{B}}{V_{L}}\;\frac{dM}{dt}=\left(k_{-L}M-k_{+L}B\right)\frac{V_{B}}{V_{M}}\;\frac{dT}{dt}=\left(k_{-T}T-k_{+L}B\right)\frac{V_{B}}{V_{T}}\;$$

where *B*, *L*, *M*, and *T* are the concentrations of CCDC in blood, liver, muscle, and tumor, respectively; *k*_+_ and *k*_-_ are constants of the rates of absorption and excretion of CCDC by various organs (*L*, *M*, or *T*); k*_E_* and *k_EL_* are constants of irreversible elimination rates of CCDC from blood and liver, respectively; and *V_B_*, *V_L_*, *V_M_*, and *V_T_* are volumes of blood, liver, muscle, and tumor. The full CCDC content in organs was calculated as *V_B_* × *B*, *V_L_* × *L*, *V_M_* × *M*, and *V_T_* × *T*. Values of organ volumes were estimated based on the data about the weight of the studied samples of the respective organs, and amounted to 1, 3, 10, and 1 mL for blood, liver, muscle, and tumor, respectively. Other constants were varied to find the best correspondence with the experimental results. The obtained system of equations was solved numerically. For verification of the hypothesis of model adequacy, the determination coefficient was used.

## Figures and Tables

**Table 1:** The best-fit values of the rate constants *k*.

Constant	k_+L_	k_-L_	k_+M_	k_-M_	k_+T_	k_-T_	k_E_	k_EL_
Value, h^−1^	0.30	1 × 10^−6^	0.07	0.67	0.05	0.20	0.48	0.55
